# Cardiovascular Side Effects of Anthracyclines and HER2 Inhibitors among Patients with Breast Cancer: A Multidisciplinary Stepwise Approach for Prevention, Early Detection, and Treatment

**DOI:** 10.3390/jcm12062121

**Published:** 2023-03-08

**Authors:** Ciro Mauro, Valentina Capone, Rosangela Cocchia, Filippo Cademartiri, Ferdinando Riccardi, Michele Arcopinto, Maie Alshahid, Kashif Anwar, Mariano Carafa, Andreina Carbone, Rossana Castaldo, Salvatore Chianese, Giulia Crisci, Roberta D’Assante, Mariarosaria De Luca, Monica Franzese, Domenico Galzerano, Vincenzo Maffei, Alberto M. Marra, Valeria Valente, Federica Giardino, Alfredo Mazza, Brigida Ranieri, Anna D’Agostino, Salvatore Rega, Luigia Romano, Sarah Scagliarini, Chiara Sepe, Olga Vriz, Raffaele Izzo, Antonio Cittadini, Eduardo Bossone, Andrea Salzano

**Affiliations:** 1 Cardiology Division, Antonio Cardarelli Hospital, Via Cardarelli 9, 80131 Naples, Italy; 2 Department of Advanced Biomedical Sciences, University of Naples Federico II, Via Sergio Pansini 5, 80131 Naples, Italy; 3 Department of Radiology, Fondazione G. Monasterio CNR-Regione Toscana, Via Moruzzi 1, 56124 Pisa, Italy; 4 Oncology Unit, Antonio Cardarelli Hospital, Via Cardarelli 9, 80131 Naples, Italy; 5 Department of Translational Medical Sciences, Federico II University, 80138 Naples, Italy; 6 The Heart Centre, King Faisal Specialist Hospital & Research Centre, Riyadh 11564, Saudi Arabia; 7 Emergency Medicine Division, Antonio Cardarelli Hospital, Via Cardarelli 9, 80131 Naples, Italy; 8 Unit of Cardiology, Department of Translational Medical Sciences, Monaldi Hospital, University of Campania Luigi Vanvitelli, 80131 Naples, Italy; 9 IRCCS SYNLAB SDN, Via Emanuele Gianturco 113, 80143 Naples, Italy; 10 Post Operative Intensive Care Division, Antonio Cardarelli Hospital, 80131 Naples, Italy; 11 Unit of Cardiology, Camerino Hospital, 62032 Macerata, Italy; 12 Department of Public Health, University Federico II of Naples, Via Sergio Pansini 5, 80131 Naples, Italy; 13 Department of General and Emergency Radiology, Antonio Cardarelli Hospital, Via Cardarelli 9, 80131 Naples, Italy; 14 Technical Nursing and Rehabilitation Service (SITR) Department, Cardarelli Hospital, 80131 Naples, Italy; 15 Department of Cardiovascular Sciences, University of Leicester, Leicester LE2 7TG, UK

**Keywords:** cardiotoxicity, cardio-oncology, prevention, breast cancer

## Abstract

Cardiovascular (CV) diseases (CVD) are a major cause of long-term morbidity and mortality affecting life expectancy amongst cancer survivors. In recent years, because of the possibility of early diagnosis and the increased efficacy of neo-adjuvant and adjuvant systemic treatments (targeting specific molecular pathways), the high percentage of survival from breast cancer led CVD to become the first cause of death among survivors. Therefore, it is mandatory to adopt cardioprotective strategies to minimize CV side effects and CVD in general in breast cancer patients. Cancer therapeutics-related cardiac dysfunction (CTRCD) is a common group of side effects of chemotherapeutics widely employed in breast cancer (e.g., anthracycline and human epidermal growth factor receptor 2 inhibitors). The aim of the present manuscript is to propose a pragmatic multidisciplinary stepwise approach for prevention, early detection, and treatment of cardiotoxicity in patients with breast cancer.

## 1. Background

Cardiovascular (CV) complications related to cancer therapy are becoming an even more significant health challenge [1]. Indeed, with the improvements in cancer treatments and survival, and with new cancer therapies continuing to emerge, cancer therapy-related cardiovascular toxicity (CTR-CVT) represents a novel and growing issue in the management of oncological patients. Therefore, a still unmet need is to prevent, identify early, and properly treat the acute, chronic, and late-onset CV toxicity of cancer treatments and to manage and improve the quality of life of cancer survivors with CV side effects [1,2].

Breast cancer is the most common malignant tumor in women, totaling about 1.4 million new diagnoses per year worldwide [3]. In recent years, early diagnosis, neoadjuvant, and adjuvant systemic treatments targeting molecular pathways have significantly improved survival rates in breast cancer [4]. On the other hand, the increased survival related to the ameliorated efficacy and effectiveness of targeted treatments led to an increase of prevalence of the cardiotoxic effects of anticancer treatment and an increase in mortality from CV causes; as a result, nowadays, CV disease (CVD) represents the first cause of death among subjects who survive breast cancer [5].

Currently, an important emerging research field of interest is focused on optimizing cancer treatment efficacy while minimizing cardiotoxicity risk [6]. To reach these results, there are several possible phases targeted for research: the basal CV risk assessment, primary prevention, early diagnosis, surveillance and monitoring, and specific treatment of CV side effects of anticancer therapies. In addition, considering the complexity of this issue, a multidisciplinary approach involving health care professionals (i.e., physicians, nurse practitioners, oncologists, cardiologists, etc.) and scientists is strongly recommended [6].

In cancer patients scheduled to receive cardiotoxic cancer therapies, baseline CV risk assessment [7], serum biomarkers [8], and CV imaging [9] techniques were validated to help clinicians for the entire management of cardiotoxicity.

The aim of the present review is to define the proper management to prevent, detect early, and treat cardiotoxicity in patients with breast cancer due to anthracyclines and/or human epidermal growth factor receptor 2 inhibitors (HER2i). Based on the expert opinion of a multidisciplinary team and on the current guidelines, a pragmatic multistep approach is presented herein.

### 1.1. Breast Cancer Chemotherapeutics: Anthracyclines and HER2i

Anthracyclines (e.g., doxorubicin and epirubicine), which are highly effective chemotherapy agents (as doxorubicin and epirubicine), are utilized in breast cancer as neoadjuvant and adjuvant treatments as well as in treatment for more advanced phases (i.e., metastasis) [10]. As a trade-off for their high efficacy in treatment, they could contribute to the development of CV side effects [11]. The risk of anthracycline-induced cardiotoxicity is dose-dependent and increases with cumulative doses. The proposed anthracycline-related mechanisms of cardiomyopathy comprise anthracycline transportation through the cardiomyocyte cell membrane, reactive oxygen species generation, damage response and repair of deoxyribonucleic acid, mitochondrial dysfunction, cardiotoxic anthracycline metabolite production, and disruption of sarcomeres [12,13].

Immunotherapies and targeted therapies implying the inhibition of human epidermal growth factor receptor 2 (HER2) signaling with either antibodies (e.g., trastuzumab and pertuzumab) or small molecule tyrosine kinase inhibitors (TKIs as lapatinib) have improved the outcomes of patients with HER2-positive (+) breast cancer [13,14]. Trastuzumab is a monoclonal antibody binding to the HER2 extracellular domain [15]; HER2 is part of the transmembrane epidermal growth factor receptor tyrosine kinases (ErbB) with a role in growth, proliferation, and repair [15]. HER2+ tumor cells display a proliferative phenotype with an augmented ability to disseminate and determine angiogenesis. HER2+ tumor cells are found in up to 30% of breast cancers [10]. Moreover, 3% to 7% of patients who received trastuzumab monotherapy developed several forms of cardiac dysfunction; this percentage is even higher when trastuzumab was administrated after prior treatment with anthracyclines [16]. Despite not being fully understood from a pathophysiological point of view, it was suggested that cardiomyocyte death happens through the direct result of ErbB2 blockade, leading to an increase in the production of reactive oxygen species [11,16,17]. (Table 1).

Recently, novel HER2i treatments were approved, particularly for patients with advanced disease and/or evidence of metastasis, even if prior anti-HER2-based regimens had been performed (e.g., trastuzumab emtansine TDM-1, trastuzumab-deruxtecan T-DXd, neratinib, and tucatinib) [18]. Specifically, TDM-1 and T-DXd could both be associated with a decrease in LVEF; however, the rate of cardiac dysfunction observed in clinical trials was similar to that of lapatinib/capecitabine regimens or antibody–drug conjugates with trastuzumab regimens [19]. Similarly, patients receiving neratinib or tucatinib experienced comparable rates of LVEF reduction, arrhythmia, and ischemic heart disease; of note, a lower rate of QTc prolongation was observed [19]. As a result, although there is less experience with these drugs in the real world, to date, there is no evidence that these agents are associated with a greater risk of cardiotoxicity [19].

Regarding the mechanism of trastuzumab-related cardiotoxicity, it differs from anthracycline toxicity [20] by determining a more favorable clinical outcome [21]. Specifically, the pathophysiology involves the signaling pathways that are interrupted by the monoclonal antibody; therefore, because the pathway restores when the trastuzumab is interrupted, the trastuzumab-related cardiotoxicity seems to be largely reversible when the treatment is withdrawn; in addition, more frequently, if an anti-LV dysfunction treatment had been started, it is possible to reintroduce trastuzumab [21].

### 1.2. Anti-Breast Cancer Treatment Cardiotoxicity

According to current guidelines, CTR-CVT includes a wide spectrum of CVD, which is grouped into a few main categories: cancer therapy-related cardiac dysfunction (CTRCD), which includes cardiac injury, cardiomyopathy, and heart failure (HF); myocarditis; vascular toxicities; arterial hypertension; cardiac arrhythmias [22].

The main clinical presentation of anthracyclines and/or HER2i cardiotoxicity is CTRCD [10], whose definition was recently univocally redefined by current guidelines on cardio-oncology from the European Society of Cardiology (ESC) [22]. Therefore, CTRCD can be divided into two categories: (i) asymptomatic CTRCD, which can be, in turn, separated into (1) mild: left ventricular ejection fraction (LVEF) ≥50% together with a decrease in global longitudinal strain (GLS) of >15% from baseline and/or an increase in cardiac biomarkers; (2) moderate: either new LVEF reduction ≥10% to an LVEF of 40–49% or new LVEF reduction <10% to an LVEF of 40–49% and either new relative decline in GLS of >15% from baseline or a new increase in cardiac biomarkers; (3) severe: new LVEF reduction to <40%; and (ii) symptomatic CTRCD (i.e., HF), which is divided into mild, moderate, severe, and very severe according to symptoms and the therapy and/or hospitalization required [22]. Notably, cardiac biomarkers occurring in the definition of CTRCD refer to pre-specified increases of both cardiac troponins (cTn, i.e., I (cTnI) and T (cTnT), considering the 99th percentile upper the reference limits) and natriuretic peptides (NPs, i.e., B-type natriuretic peptide (BNP) ≥ 35 pg/mL and N-terminal pro-B-type natriuretic peptide (NT-proBNP) ≥ 125 pg/mL) or a new significant rise from baseline above the biological and analytical fluctuation of the assay employed [22,23].

## 2. A Stepwise Approach for Prevention and/or Early Detection of Cardiotoxicity

### 2.1. General Principles

A close and early collaboration between cardiologists, oncologists, physicians, and radiotherapist oncologists is recommended to improve lifetime CV health and to ward off unnecessary cessations of cancer treatment [24].

Screening for pre-existing CV risk factors in all patients with breast cancer and treatment of the recognized CV risk factors according to current European Society guidelines is recommended [24]. In fact, the assessment of conventional CV risk factors and optimal treatment of CVD, according to the current guidelines, should be routinely applied for all patients prior to, during, and after receiving cancer therapy [25].

### 2.2. Baseline CV Risk Assessment

As first a step, CV risk factors should be screened in all patients with breast cancer; in addition, the European Society of Cardiology (ESC)’s guidelines on cardio-oncology, drafted along with the European Hematology Association, the European Society for Therapeutic Radiology and Oncology, and the International Cardio-Oncology Society (ICOS) society of Cardiology strongly recommend monitoring CV safety when using therapies with known CV toxicities (i.e., mediastinal and left-sided chest radiation, chemotherapy, and targeted agents which are proven to affect the heart and vascular system) [24]. Therefore, clinicians involved in the cardio-oncology setting are required to find oncologic patients who are at increased risk to develop CTR-CVT, defining the so-called “baseline CV risk” [7].

The ESC guidelines recommend performing a risk stratification of possible CV toxicity before starting potentially cardiotoxic anticancer drugs in all subjects with breast cancer [22]. With this aim, the Cardio-Oncology Study Group from the Heart Failure Association (HFA) of the ESC, in collaboration with the ICOS, developed the “Baseline CV risk stratification proformas”, which is able to assess the baseline CV risk in cancer patients ongoing several classes of cancer therapies, as anthracyclines and HER2-targeted therapies are known to cause a range of CV toxicities [7] including, but not limited to, left ventricular disfunction (LVD) and HF [7]. They suggest early attainment of the baseline CV risk assessment proformas in all patients expected to be treated with anthracycline or HER2i to avoid cancer therapy postponement and confirm that HER2i can be safely started. Obviously, in emergency settings, guideline-based cancer therapy should be initiated immediately, and the baseline CV risk assessment proformas can be postponed until after the patient is clinically stable [7].

Baseline CV risk assessments should be considered by the treating oncologist or by a cardiologist, if appropriate. The HFA-ICOS baseline risk assessment enables risk stratification for patients who are going to start potential cardiotoxic treatment, sorting them into four risk categories: low, moderate, high, and very high [22].

Low-risk patients can follow routine oncology follow-ups and eventually be referred to cardio-oncology if a CTR-CVT emerges. Moderate-risk patients can benefit from closer oncology follow-ups and a referral to cardio-oncology if CTR-CVT develops. On the other end, for high- and very high-risk patients, a cardiology referral is recommended immediately, as there is a need to balance the risk versus benefit of cancer treatment and to consider cardioprotective strategies [22].

Two main sets of factors (i.e., patient-related and therapy-related) contribute to baseline CV risk. Risk assessment should include clinical history (including history of CVD or of previous cancer treatments and CV risk factors), physical examination (including vital sign measurements), and general blood tests (including fasting plasma glucose/HbA1c, kidney function, and lipid profile) [22].

In all patients starting cancer therapy, it is recommended to perform an electrocardiogram (ECG, including measurement of heart rate QTc [24]) as part of their baseline risk assessment; if abnormalities are detected (i.e., advanced conduction disease, Q waves in two or more contiguous leads, left ventricular (LV) hypertrophy, new atrial fibrillation/flutter, QTc prolongation, etc.), then referral to a cardiologist is recommended [22].

#### 2.2.1. Circulating Cardiac Biomarkers in Baseline CV Risk Assessment

Cardiac biomarkers (NPs or cTn) may be considered at baseline for risk stratification using the same assay that will be used during follow-up measurements [13]. What comprises routine use of cardiac biomarkers (cTn (cTnI or cTnT), BNP, or NT pro-BNP) for patients at risk because of cardiotoxic chemotherapy is not well-defined [8]. Moreover, according to current guidelines, the baseline measurement of cardiac serum biomarkers is recommended in all subjects undergoing cancer therapy with a demonstrated CTRCD (i.e., breast cancer patients undergoing anthracycline or HER2i) [22]. About specific chemotherapy regimens, it should be noted that the use of cardiac serum biomarkers to diagnose CTRCD is less detailed during anti-HER2 treatments than during anthracycline treatments [22]. It was suggested that an elevated cTn in patients treated with anthracyclines and eligible to be treated with trastuzumab is useful to distinguish breast cancer patients who are higher risk to develop trastuzumab-induced CTRCD [8]. However, when comparing NPs vs cTn, NT pro-BNP appears to have a higher sensitivity than troponin in detecting new LVD during trastuzumab treatment [8].

#### 2.2.2. Imaging Tests in Baseline CV Risk Assessment

In patients scheduled to have an anticancer treatment regimen associated with a risk of HF or LVD occurrence, echocardiography baseline evaluation (with a particular focus on LVEF and diastolic function) is recommended [13,24,26]. According to current guidelines, baseline comprehensive transthoracic echocardiography (TTE) is recommended in all patients with cancer at high and very high risks of CV toxicity prior to cancer treatment. The same imaging modality and method used to determine LVEF should be used during cancer therapy and during follow-up as well as after treatment; in addition, the digital images collected to determine LVEF should be compared with previous ones to diminish interobserver variability [13,24,26] and, whenever feasible, the use of three-dimensional (3D) TTE should be preferred for LVEF determination due to its enhanced reproducibility and accuracy [26]. Moreover, it is advisable to perform myocardial strain imaging at baseline, as it could be a more sensitive parameter of LVD, with a relative percentage decrease in GLS of >15% from baseline being very expected to be anomalous [26]. In selected patients, other CV complementary tests to consider include the following: (i) cardiac magnetic resonance (CMR), whose use is spread in breast cancer patients with breast prothesis (that often makes inadequate echocardiographic acoustic windows); (ii) coronary computed tomography angiography (CCTA), which is often the imaging method of choice to exclude coronary involvement in young breast cancer patients given their low pre-test probability of coronary arteries diseases; (iii) cardiopulmonary exercise testing (CPET), currently employed to assess pre-operative risk stratification (but more validated in patients with colon, lung, and rectal cancers than in breast cancer) [22].

Therefore, according to the evaluated baseline CV risk of breast cancer patients, preferably following HFA-ICOS risk stratification tools, validated surveillance protocols and, in selected cases, cardioprotective strategies will be proposed depending on the different anticancer drugs in use (anthracycline or HER2i) [7].

### 2.3. Primary Prevention

The primary prevention approach can be contemplated for HF, ischemia, arrhythmia, hypertension, and thromboembolism. However, most of the literature and recommendations available are focused on prevention of HF in patients treated with anthracyclines; therefore, specific strategies on the primary prevention for specific CV side effects represents a gap in the evidence in need of further research [22,27].

In general, the management of CV risk factors, in agreement with the 2021 ESC guidelines on CVD prevention in clinical practice, is recommended before, during, and after cancer therapy, without delaying cancer treatments [22].

#### 2.3.1. Primary Prevention of CTRCD

With the aim of focusing the attention of clinicians on the prevention of the development of HF, patients receiving anthracycline or HER2i should be defined as patients at stage A of HF (i.e., subjects at risk of HF but with no evidence of structural heart disease or symptoms of HF) [28].

In patients with normal LVEF and CV risk factors, preventive therapy with angiotensin-converting enzyme inhibitors (ACE-Is) or angiotensin receptor blockers (ARBs) (if intolerant to ACE-Is) and/or selected beta-blockers (BBs) may be considered to diminish the possible development of cardiotoxicity [24,26,29,30].

In particular, according to current guidelines, primary prevention strategies with ACE-Is/ARBs and BBs (preferably carvedilol) should be referred to high- and very high-risk patients treated with anthracyclines and/or HER2i [22].

Amongst other cardioprotective strategies in patients on anthracyclines, administration of cardioprotective agents such as dexrazoxane, an intracellular iron-chelating agent, or the liposomal formulation of anthracyclines should be considered in patients with cancer at high and very high CV toxicity risks [22].

In addition to CTRCD primary prevention, according to current guidelines, in breast cancer patients at high and very high CV toxicity risks, statins should be considered for primary prevention [22].

As non-pharmacological interventions, a healthy diet, cessation of smoking, aerobic physical exercise (i.e., light walking or cycling), and weight control should be strongly encouraged to reduce the risk of cardiotoxicity [31,32].

#### 2.3.2. Primary Prevention of Thromboembolic Events

Almost 1% of women with breast cancer experience venous thromboembolism (VTE) within 2 years after the diagnosis, more often in the first 6 months. Older age, increasing numbers of comorbidities, and advancing stages were associated with higher risks of VTE occurrence. The major risk factor is metastatic cancer at the time of diagnosis (about a sixfold increased risk of VTE compared with localized cancer), whereas the histological subtype is not a predictor of VTE [33].

The individual risk of chemotherapy associated VTE is usually assessed through validated scores [34] (i.e., KHORANA) using baseline clinical and laboratory variables.

In hospitalized patients with cancer, the American Society of Hematology (ASH) guideline panel suggests performing primary prevention over no thromboprophylaxis, preferably employing low molecular weight heparin (LMWH) rather than unfractionated heparin (UFH) or mechanical thromboprophylaxis [35]. However, these are conditional recommendations (i.e., with low or very low certainty in the evidence of their effects). Consistently, ESC guidelines also prescribe preventive LMWH for the primary prevention of VTE in patients who are hospitalized and affected by cancer or those with protracted bedrest with no active bleeding or other known contraindications [22].

For ambulatory patients, thromboprophylaxis should be recommended according to the individual’s score-based risk of thromboembolism [35,36]. Therefore, in ambulatory patients receiving systemic therapy displaying low risk for thrombosis, they recommend no thromboprophylaxis (over neither parenteral nor oral thromboprophylaxis); in ambulatory patients at intermediate risk for thrombosis, the ASH guideline panel advises either performing thromboprophylaxis with apixaban or rivaroxaban as a direct oral anticoagulant (DOAC) or not performing thromboprophylaxis at all [35]. Finally, in ambulatory patients with cancer at high risk for VTE, either LMWH or a DOAC (apixaban or rivaroxaban) are suggested over no thromboprophylaxis [35], provided there are no significant contraindications, which is also recommended according to current ESC guidelines [22].

For cancer patients with a central venous catheter (CVC), the ASH guideline panel suggests not using either oral or venous thromboprophylaxis [35].

In conclusion, the use of thromboprophylaxis should be based on individual benefit–risk estimations.

### 2.4. Validated Surveillance Protocols

Based on the baseline CV estimated risk, several surveillance approaches have been proposed based both on patient and cancer treatment characteristics [8,9,22].

The ESC guidelines define the exact timing of monitoring after the baseline evaluation (i.e., before starting cancer treatment), of serum biomarkers and echocardiography during treatment with anthracycline and HER2-targeted therapies [8,9].

Table 2 and Table 3 show a summary of the recommended biomarker and echocardiographic surveillance regimens during and after anthracycline therapy and HER2-targeted therapies, according to ESC recommendations, after the assessment of the baseline CV risk of cardiotoxicity using the HFA-ICOS tool [8,9].

### 2.5. Secondary Prevention

Secondary prevention is addressed to patients with pre-existing CVD, including previous CTR-CVT, and/or who have experienced new CTR-CVTs during cancer treatment. CVD can occur before, during, and after cancer treatment; there are two main domains in which secondary prevention is currently recommended: CTRCD and VTE [22].

#### 2.5.1. Patients with CTRCD

Subjects with asymptomatic LVEF impairment fulfilling the criteria of cardiotoxicity are to be considered to be stage B HF subjects (i.e., patients with structural heart disease but who have never experienced symptoms of HF), mainly if associated with an increase in NPs [37]. Based on the degree of LVEF reduction, guideline-based HF therapies should be considered with an ACE-I (or ARB) and BB [38]. At present, if early signs of subclinical myocardial dysfunction are detected through TTE-based GLS surveillance, then there is no evidence to implement specific cardioprotection measures. GLS is more sensitive than LVEF when identifying early cardiotoxicity; however, according to present evidence, cancer treatment should not be stopped, interrupted, or have its doses diminished because of new evidence of isolated GLS reduction [13,39,40,41]. Regarding serum biomarkers, the increase in NP levels further supports the diagnosis of HF; on the other hand, cTn elevation without evidence of LVD does not induce permanent discontinuation of anticancer treatment. Careful evaluation and the addition of cardioprotective therapy, together with strict follow-ups, should be considered [13,24].

Symptomatic patients displaying an impairment in LVEF are classified as stage C HF and should be treated according to current ESC guidelines for HF [24,42]. In this scenario, cardiologists, and oncologists should conference with each other, preferably referring to a cardio-oncology specialist service, to decide on the parameters of the interruption (i.e., necessity, duration, etc.) of cancer therapy with the aim of balancing the risk vs. the benefit of maintaining the prior drug regimen according to the severity of LVD, signs and symptoms of HF, cancer prognosis, and efficacy of the oncologic treatment [13].

##### Secondary CTRCD Prevention with Anthracycline

According to current ESC guidelines, during anthracycline chemotherapy, HF treatment is recommended for patients who have experienced symptomatic CTRCD or with evidence of asymptomatic moderate or severe CTRCD. Patients with LVEF ≥ 50% and a significant decline in GLS with a cTn elevation > upper normal limit (ULN), ACE-I/ARB, and/or BBs should be considered (although with a lower class of recommendation if the rise of cardiac biomarkers regards only NPs) [22].

In patients who develop symptomatic severe or moderate CTRCD, the discontinuation and/or transitory interruption of anthracyclines are recommended, respectively; if symptomatic mild CTRCD occurs, then a multidisciplinary approach before interruption is required. The provisional interruption of anthracycline chemotherapy is also recommended in patients with asymptomatic moderate or severe CTRCD; however, the continuation of anthracycline chemotherapy is recommended in asymptomatic patients who have LVEF ≥ 50% [22].

In all patients with moderate or mild symptomatic CTRCD or with moderate or severe asymptomatic CTRCD, the choice of recommencing oncologic therapy requires a multidisciplinary agreement. If a further anthracycline regimen is planned, then the use of dexrazoxane and/or liposomal doxorubicin may be considered in patients with moderate or severe symptomatic or asymptomatic CTRCD to reduce the risk of further CV toxicity [22].

##### Secondary CTRCD Prevention with HER2-Targeted Therapy

HF therapy is recommended for patients on HER2i with symptomatic moderate-to-severe CTRCD with LVEF < 50%, mild symptomatic CTRCD, or asymptomatic severe CTRCD. BBs and ACE-I/ARB are recommended in patients who develop asymptomatic and moderate (LVEF 40–49%) CTRCD; it is less-recommended for patients who have LVEF ≥ 50% but develop a significant decline in GLS or a new cTn or NPs rise while continuing HER2-targeted therapy [22].

**Figure 1:**
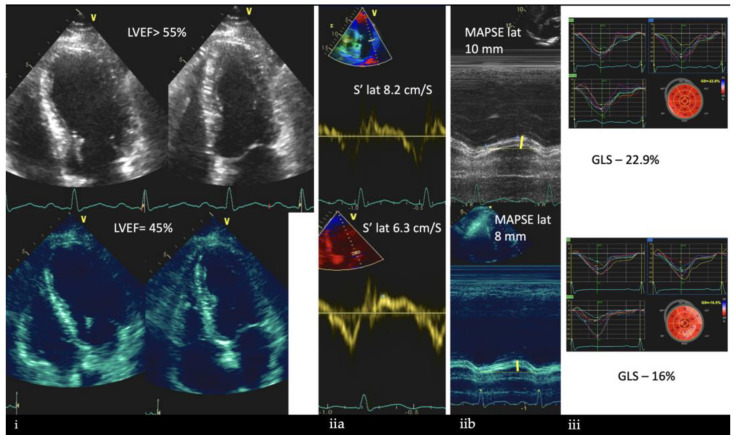
An example of detection and management of left ventricular disfunction (LVD) due to herceptin (anti HER2i) treatment in the case of a 59-year-old woman with breast cancer. In the upper panel, LV ejection fraction (EF), lateral S’, mitral annular plane systolic excursion (MAPSE), and global longitudinal strain (GLS) before chemotherapy are reported. All values were in the normal range. The lower panel depicts the same parameters after two sessions of chemotherapy, showing an impairment in cardiac function. Therefore, the patient started heart failure therapy (b/blockers and ACE-inhibitors); after four months, we observed a full recovery of the LVD. The detection of LVD was determined by the assessment of LV function in 2D using (from left to right): (**i**) EF through the modified Simpson’s method of discs, acquiring LV volumes from apical four- and two-chamber views; (**ii**) linear methods from apical four chamber view via: (**iia**) tissue Doppler imaging (TDI) of the mitral annulus, with sample volume placed 1 cm at the lateral annulus of mitral valve and (**iib**) M-mode on the lateral annulus of mitral valve; (**iii**) GLS, derived from speckle tracking and analyzed by post-processing the apical images of the LV as a myocardial deformation analysis reflecting the sub-endocardial, longitudinally oriented fibers’ function.

It is recommended to discontinue and/or temporarily interrupt HER2i in patients who develop moderate or severe symptomatic CTRCD or severe asymptomatic CTRCD; the choice to reestablish HER2i should be reached based on interdisciplinary judgment after the improvement of LV function and the resolution of symptoms. Discontinuation of HER2-targeted therapy is not recommended for patients who experience mild CTRCD, and it should be considered in patients who experience asymptomatic moderate CTRCD. More frequent cardiac monitoring should be accomplished in both cases [22] (Figure 1).

#### 2.5.2. Secondary Prevention of VTE

Even if less frequent than in other cancers, breast cancer could also be associated with VTE (in almost 1% of cases). DOAC can be considered as a possible alternative to LMWH for cancer-associated VTE. Therefore, according to the ESC guidelines for symptomatic or incidental VTE occurring in patients with cancer without contraindications (i.e., unoperated gastrointestinal or genitourinary malignancies, history of recent bleeding or within 7 days of major surgery, platelet count < 50,000/μL, creatinine clearance < 15 mL/min, or gastrointestinal comorbidities), apixaban, edoxaban, or rivaroxaban are recommended. As an alternative, LMWH is recommended for the treatment of symptomatic or incidental VTE in patients with cancer with platelet counts > 50,000/μL; the LMWH dose may be halved if the platelet count is 25,000–50,000/μL, and administration should be decided by clinicians [22].

The minimal duration of anticoagulation is 6 months, and prolonging anticoagulation therapy beyond 6 months should be considered in the presence of an active malignancy, advanced cancer, or chemotherapy use [22].

For catheter associated VTE, ESC guidelines recommend continuing anticoagulation for a minimum of 3 months; if the catheter remains, then a longer period is suggested [22].

**Figure 2:**
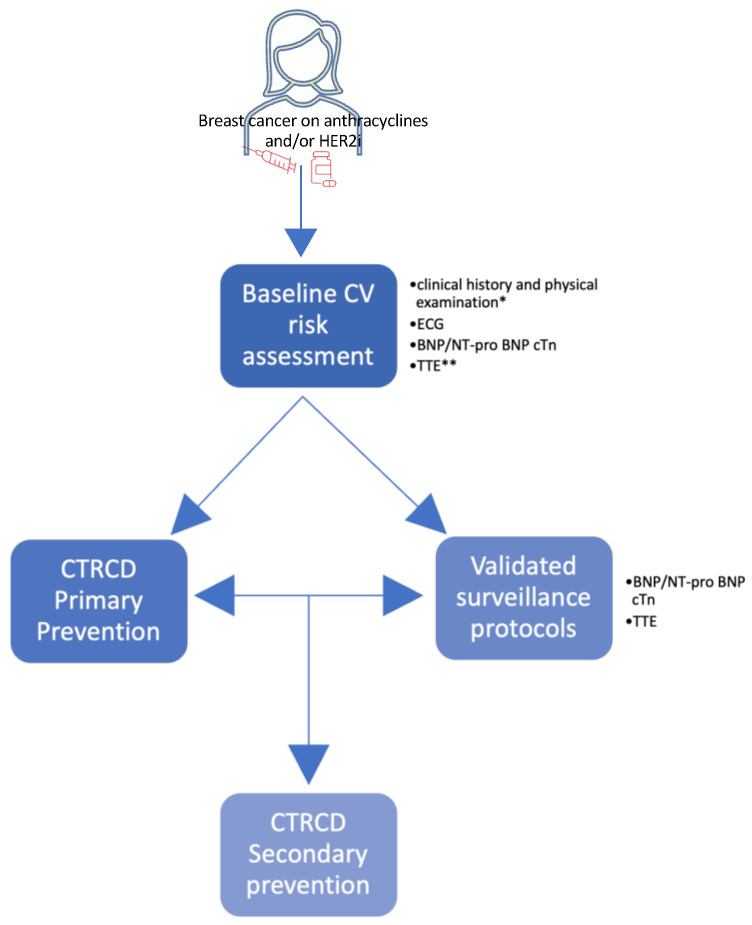
A stepwise multidisciplinary approach to management of cardiotoxicity in breast cancer patients undergoing anthracycline and/or HER2i treatment. * Including vital sign measurements, general blood tests including fasting plasma glucose/HbA1c, kidney function, and lipid profile. ** In selected cases. Consider other CV complementary tests in selected patients: cardiac magnetic resonance (CMR), coronary computed tomography angiography (CCTA), or cardiopulmonary exercise testing (CPET). Abbreviations: BNP, B-type natriuretic peptide; cTn, cardiac troponins; CTRCD, cancer-therapeutics-related cardiac dysfunction; HER2i, HER2 inhibitors; NT-proBNP, N-terminal pro B-type natriuretic peptide; TTE, transthoracic echocardiography.

A “stepwise multidisciplinary approach” to cardiotoxicity management in patients with breast cancer and undergoing anthracycline and/or HER2i treatment is depicted in Figure 2 [22].

## 3. Long Term Follow-Up

According to ESC guidelines, breast cancer survivors treated with anthracycline and HER2i need CV risk assessments yearly, including ECG and NPs, to check for clinical signs and symptoms, monitor blood pressure, cholesterol, and glycated hemoglobin, and manage CV risk factors. Further, at 5 years after therapy, a new CV toxicity risk stratification is recommended to organize long-term follow-up [22].

Echocardiographic exams should be considered at years 1, 3, and 5 after ending cardiotoxic oncologic treatment and every 5 years after that time, in particular in asymptomatic very high- and early high-risk breast cancer survivors [22].

HF in cancer survivors should be treated according to the 2021 ESC guidelines on acute and chronic HF. ACE-I/ARB and/or BBs are recommended in patients who develop moderate asymptomatic CTRCD; if there is evidence of mild asymptomatic CTRCD (LVEF > 50% but a new decrease in GLS and/or cardiac serum biomarker increase), then ACE-I/ARB and/or BBs may be started [22,42].

## 4. Gaps in Evidence and Future Perspectives

Despite an increasing body of data available in the last years, several gaps in evidence remain; as highlighted by the most recent ESC guidelines [22], additional efforts are needed to better clarify the role of cardio-oncology services and programs as well as the role of focused cardio-oncology rehabilitation. In addition, regarding research trials, it would be promising to see that cardio-oncology teams become more involved in trial design as well, as it is still necessary to adopt a common shared terminology about CV toxicity as well as a common CV side effect-monitoring strategy. Future research is needed regarding the proper prevention, diagnosis, and management of CV toxicity. Finally, there is a lack of evidence about the development of possible CV diseases in long-term cancer survivors, with a need for focused research on CV-preventive and management strategies for long-term survivors.

## 5. Conclusions

CVD is a major cause of long-term, non-cancer-related morbidity and mortality amongst cancer survivors, limiting their life expectancy. Thanks to the increased efficacy and efficiency of treatments currently available, CVD is now the main motive of death among female survivors of breast cancer.

Therefore, cardioprotective strategies have a pivotal role for anthracycline- and trastuzumab-treated patients, which is supported by strong evidence from current guidelines.

A pragmatic stepwise approach to prevent main CV side effects and to manage the cardiotoxicity of cancer treatment in breast cancer is suggested; in addition, validated surveillance protocols for both anthracyclines and HER2i are available based on current guidelines and recommendations, and they are strongly advised to early detect potential cardiotoxicity. Third, strategies to manage CV side effects are available to guarantee longer life expectancy and a higher quality of life to breast cancer patients. Finally, a long-term surveillance strategy is suggested in high- and very high-risk patients.

## Figures and Tables

**Table 1:** Comparison of the main characteristics of anthracyclines and HER2i.

Drug	Anthracyclines	HER2i
**Agents**	Doxorubicin, epirubicine	Antibodies: trastuzumab, pertuzumab TKIs: lapatinib
**Type of tumor**	Breast cancer, sarcoma, lymphoma, pediatric leukemia	HER2 positive breast cancer *, stomach/gastroesophageal junction -HER2 positive adenocarcinoma
**Type of administration**	Endovenous **	Endovenous
**Metabolism**	Hepatic	Hepatic
**Elimination**	Biliary and faecal excretion ***	Biliary and faecal excretion
**Type of cardiotoxicity**	CTRCD (dose dependent)	CTRCD (not dose dependent)
**Mechanism of cardiotoxicity**	DNA double-stranded breaks Defective mitochondrial biogenesis Increase in ROS	Cardiomyocyte death through the direct result of ErbB2 (HER2) blockade Increase in ROS
Common non-cardiotoxic adverse effects ****	Sepsis Bone marrow suppression Anorexia Nausea, vomiting, mucositis/stomatitis, diarrhea Alopecia	Interstitial lung disease, dyspnoea, cough, epistaxis Upper respiratory tract infection Bone marrow suppression Hypokalemia, anorexia, headache, dizziness, musculoskeletal pain, fatigue, fever Nausea, vomiting, diarrhea, abdominal pain, constipation, stomatitis, dyspepsia, increased transaminases Alopecia

* HER2+ tumor cells found in up to 30% of breast cancers; it can be employed as monotherapy or after prior treatment with anthracyclines; risk of development of HER2-targeted cardiomyopathy increases in patients previously treated with anthracyclines; ** intravesical instillation for treatment of superficial bladder cancer or as prophylaxis to prevent tumor recurrence after transurethral resection; *** 10% of the dose is excreted renally; **** intravesical treatment may cause local reactions (chemical cystitis) (e.g., dysuria, urinary frequency, nocturia, strangury, hematuria, and bladder wall necrosis); AML, acute myeloid leukemia; CTRCD, cancer therapeutics-related cardiac dysfunction, DNA, deoxyribonucleic acid; HER2, human epidermal growth factor receptor 2; HER2i, human epidermal growth factor receptor 2 inhibitor; MDS, myelodysplasia; ROS, reactive oxygen species; TKIs, small molecule tyrosine kinase inhibitors; Top, topoisomerase.

**Table 2:** Biomarker (circulating and imaging)-based surveillance during and after anthracycline therapy.

Baseline Risk of Cardiotoxicity	Type of Surveillance	During Chemotherapy	Following Chemotherapy
**Low**	BNP/NT-proBNP cTn	Baseline Every 2 cycles Every 2 cycles in patients receiving a cumulative lifetime cycle of 250 mg/m^2^ doxorubicin or equivalent ^a^	Within 3 months after final cycle Within 3 months after final cycle in patients receiving a cumulative lifetime cycle of 250 mg/m^2^ doxorubicin or equivalent ^a^
	Echocardiography	Baseline Following cycle completing cumulative lifetime dose of 250 mg/m^2^ doxorubicin or equivalent ^a^	12 months after final cycle
**Moderate**	BNP/NT-proBNP cTn	Baseline Every 2 cycles	Within 3 months after final cycle
	Echocardiography	Baseline Following cycle completing cumulative lifetime cycle of 250 mg/m^2^ doxorubicin or equivalent ^a^	12 months after final cycle
**High and Very High**	BNP/NT-proBNP cTn	Baseline Before every cycle	3 and 12 months after final cycle
	Echocardiography	Baseline Every 2 cycles	Within 3 months after final cycle 12 months after final cycle

Abbreviations: BNP, B-type natriuretic peptide; cTn, cardiac troponin; CV, cardiovascular; NT-pro BNP, N-terminal B-type natriuretic peptide; cycle of chemotherapy infusion. ^a^ 100 mg/m^2^ of doxorubicin (CV toxicity dose ratio 1) equivalent to: 125 mg/m^2^ of epirubicin (CV toxicity dose ratio 0.8); 167 mg/m^2^ daunorubicin (CV toxicity dose ratio 0.6); 9.5 mg/m^2^ mitoxantrone (CV toxicity dose ratio 10.5); 20 mg/m^2^ idarubicin (CV toxicity dose ratio 5).

**Table 3:** Biomarker and echocardiographic surveillance during and after HER2-targeted therapies.

	Baseline Risk of Cardiotoxicity	Type of Surveillance	During Chemotherapy	Following Chemotherapy
Early invasive HER2+ breast cancer with neoadjuvant or adjuvant trastuzumab ^a^	Low	BNP/NT-proBNP cTn	Baseline Every 3 months	12 months after final cycle
		Echocardiography	Baseline Every 3 months (every 4 months if normal assessment after 3 months and asymptomatic)	Within 12 months after final cycle
	Moderate	BNP/NT-proBNP cTn	Baseline Every 3 months	12months after final cycle
		Echocardiography	Baseline Every 3 months	within 12 months after final cycle
	High and Very High	BNP/NT-proBNP cTn	Baseline Every cycle 2 – 3 cycles	3 months and 12 months after final cycle
		Echocardiography	Baseline Every 3 months Every 2 or 3 cycles (depending on absolute risk and local availability)	Within 12 months after final cycle
	Low and Moderate post AC prior to starting anti HER2	cTn	Baseline	
Metastatic HER2+ breast cancer with long-term HER2-targeted therapies ^b^	Low and Moderate	Echocardiography	Every 3 months in year 1; if asymptomatic and no CTR-CVT every 6 months	
	High and Very High		Every 2 or 3 cycles depending on the absolute risk and local availability	

Abbreviations: AC, anthracycline, BNP, B-type natriuretic peptide; cTn, cardiac troponin; CV, cardiovascular; HER2, human epidermal growth factor receptor 2; NT-pro BNP, N-terminal B-type natriuretic peptide; cycle, chemotherapy infusion. a Neoadjuvant trastuzumab or trastuzumab and pertuzumab. b Long-term trastuzumab, trastuzumab and pertuzumab, or trastuzumab emtansine.

## Data Availability

The data are available from the corresponding author on reasonable request.

## Abbreviations:

3D	three-dimensional
ACE-I(s)	angiotensin-converting enzyme inhibitor(s)
ASH	American Society of Hematology
ARB(s)	angiotensin receptor blocker(s)
BB(s)	beta-blocker(s)
BNP	B-type natriuretic peptide
CCTA	coronary computed tomography angiography
CMR	cardiac magnetic resonance
CPET	cardiopulmonary exercise testing
cTn	cardiac troponins
cTnI	cardiac troponin I
cTnT	cardiac troponin T
CTRCD	cancer therapeutics-related cardiac dysfunction
CTR-CVT	cancer therapy-related cardiovascular toxicity
CV	cardiovascular
CVD	cardiovascular diseases
DOAC	direct oral anticoagulants
ESC	European society of cardiology
GLS	global longitudinal strain
HER2	human epidermal growth factor receptor 2
HER2i	human epidermal growth factor receptor 2 inhibitors
HF	heart failure
HFA-ICOS	Heart Failure Association—International Cardio-Oncology Society
ICOS	International Cardio-Oncology Society
LMWH	low molecular weight heparin
LV	left ventricular
LVD	left ventricular dysfunction
LVEF	left ventricular ejection fraction
NPs	natriuretic peptides
NT-proBNP	N-terminal pro B-type natriuretic peptide
TKIs	small molecule tyrosine kinase inhibitors
TTE	transthoracic echocardiography
UFH	unfractioned heparin
ULN	upper normal limit
VTE	venous thrombosis and venous thromboembolism
